# Glycosylation Alterations in Multiple Sclerosis Show Increased Proinflammatory Potential

**DOI:** 10.3390/biomedicines8100410

**Published:** 2020-10-13

**Authors:** Ana Cvetko, Domagoj Kifer, Olga Gornik, Lucija Klarić, Elizabeth Visser, Gordan Lauc, James F. Wilson, Tamara Štambuk

**Affiliations:** 1 Faculty of Pharmacy and Biochemistry, University of Zagreb, 10000 Zagreb, Croatia; acvetko@pharma.hr (A.C.); dkifer@pharma.hr (D.K.); ogornik@pharma.hr (O.G.); 2 Genos Glycoscience Research Laboratory, 10000 Zagreb, Croatia; 3 MRC Human Genetics Unit, Institute of Genetics and Molecular Medicine, University of Edinburgh, Edinburgh EH4 2XU, UK; klaric.lucija.klaric@gmail.com (L.K.); Jim.Wilson@ed.ac.uk (J.F.W.); 4 The Institute of Applied Health Sciences, MRC University of Aberdeen, Aberdeen AB25 2ZD, UK; e.visser@abdn.ac.uk; 5 Centre for Global Health Research, Usher Institute, University of Edinburgh, Edinburgh EH8 9AG, UK

**Keywords:** immunoglobulin G in inflammation, multiple sclerosis, N-glycosylation, plasma glycoproteins, biomarkers

## Abstract

Multiple sclerosis (MS) is an inflammatory autoimmune disorder affecting the central nervous system (CNS), with unresolved aetiology. Previous studies have implicated N-glycosylation, a highly regulated enzymatic attachment of complex sugars to targeted proteins, in MS pathogenesis. We investigated individual variation in N-glycosylation of the total plasma proteome and of IgG in MS. Both plasma protein and IgG N-glycans were chromatographically profiled and quantified in 83 MS cases and 88 age- and sex-matched controls. Comparing levels of glycosylation features between MS cases and controls revealed that core fucosylation (*p* = 6.96 × 10^−3^) and abundance of high-mannose structures (*p* = 1.48 × 10^−2^) were the most prominently altered IgG glycosylation traits. Significant changes in plasma protein N-glycome composition were observed for antennary fucosylated, tri- and tetrasialylated, tri- and tetragalactosylated, high-branched N-glycans (*p*-value range 1.66 × 10^−2^–4.28 × 10^−2^). Classification performance of N-glycans was examined by ROC curve analysis, resulting in an AUC of 0.852 for the total plasma N-glycome and 0.798 for IgG N-glycome prediction models. Our results indicate that multiple aspects of protein glycosylation are altered in MS, showing increased proinflammatory potential. N-glycan alterations showed substantial value in classification of the disease status, nonetheless, additional studies are warranted to explore their exact role in MS development and utility as biomarkers.

## 1. Introduction

Multiple sclerosis (MS) is a disease affecting the central nervous system (CNS), and is characterized by inflammation and demyelination that eventually lead to axon degeneration and physical disability [1]. It affects women three times more often than men [2], with an average age of onset around 30 years [3,4]. MS prevalence has been rising across North America and Europe in the past decades, where persistent geographical risk gradients have been documented, displaying its unusually high prevalence in Scotland, in particular, on the Northern Isles of Orkney and Shetland [5,6]. Therefore, a considerable interest in investigating MS aetiology on these two populations has been shown [7]. MS is essentially an inflammatory autoimmune disorder, a multifactorial disease heavily influenced by both genetic and environmental factors; however, the exact mechanism underlying its development is not yet fully understood [8,9]. Additional evidence, proving that an interplay between genetics, immunity and environment is determining MS predisposition, has been provided in recent large genome wide association studies (GWAS). These studies identified hits in multiple loci containing genes coding for various molecules involved in immune processes and environmental risk factors, such as enzymes involved in vitamin D metabolism [10,11].

Grigorian and co-workers [12] have shown that genetic and environmental factors appear to only slightly increase the risk of MS individually, but when combined, they form epistatic interactions to dysregulate one of the most frequent posttranslational modifications—N-glycosylation. These interactions can lead to the decrease in N-glycan branching on T cells, resulting in defective self-tolerance, repression of their growth, hyperactivity of the innate immune system and increased sensitivity of neurons to death, contributing to neurodegeneration and MS development. N-glycosylation is the most complex posttranslational modification and denotes a highly regulated, enzyme-mediated attachment of complex sugars to targeted proteins. Addition of different N-glycans to proteins increases both their structural and functional diversity. A perfect example is immunoglobulin G (IgG), whose effector functions are modulated by the attachment of glycans with specific monosaccharide moieties. For instance, the presence of sialic acid enhances IgG’s anti-inflammatory potential, while bisecting N-acetylglucosamine (GlcNAc) increases its proinflammatory activity. Furthermore, the presence of core fucose creates steric disruption of binding between IgG Fc fragment and FcγRIIIa receptor, resulting in inefficient activation of antibody-dependent cellular cytotoxicity (ADCC) [13,14,15,16]. Moreover, another GWAS, examining the associations between IgG N-glycosylation traits and various autoimmune disorders, identified hits in multiple genetic loci encompassing genes involved in N-glycan biosynthesis, including the *FUT8* gene, which encodes the alpha-(1,6)-fucosyltransferase responsible for the attachment of core fucose to the complex N-glycans [17]. One particular *FUT8* allele variant (rs8007846) was previously associated with the regulation of blood glutamate levels in multiple sclerosis patients [18], further enhancing the implications for N-glycosylation’s role in the MS development.

Research studies focused on alterations of IgG or total plasma protein N-glycome in MS are scarce. One study examined IgG glycosylation pattern in cerebrospinal fluid (CSF) [19], while another tackled MS-related IgG1 glycosylation changes in serum and CSF [20]. In general, the presence of oligoclonal IgG in CSF is considered to be a hallmark of MS [21]. The aforementioned study by Wuhrer et al. showed that N-glycosylation differs between CSF- and serum-derived IgG1. Moreover, CSF-derived IgG1 N-glycosylation differed between subjects and controls, but that was not the case for the glycosylation profile of the serum-derived IgG1.

Since N-glycosylation regulates the function of some key members of the immune system, that are, at the same time, involved in the MS pathophysiology, examining disease-related alterations of glycosylation shows a great potential in the biomarker field. Nonetheless, hardly any study has dealt with these complex glycosylation changes in MS, whose elucidation could assist in the process of determining the exact underlying pathophysiological mechanism and possibly provide novel biomarkers or therapeutic targets. For that reason, we aimed to investigate the potential role of N-glycosylation in multiple sclerosis by analysing IgG and plasma protein N-glycome composition in MS subjects and matching controls, recruited through Northern Isles Multiple Sclerosis Study (NIMS) from one of the most MS-prevalent regions of the world.

## 2. Experimental Section

### 2.1. Study Population

Study participants from the Orkney and Shetland Isles were recruited through the Northern Isles Multiple Sclerosis (NIMS) Study, which aimed to investigate genetic and other factors contributing to the risk of developing MS. A prevalence study from the year 2012 showed that Orkney has the highest prevalence of MS in the world, with the Shetland Isles following closely [5]. Our study population included 171 participants in total—83 multiple sclerosis cases and 88 age- and sex-matched controls [7]. Case-group included subjects with four different disease courses: relapsing remitting (RRMS), primary progressive (PPMS), secondary progressive (SPMS) with relapses, and secondary progressive (SPMS) without relapses. Basic characteristics of the study participants are given in Table 1.

### 2.2. Ethical Statement

The Genetics of Multiple Sclerosis in the Northern Isles of Scotland study (Chief Investigator J.F.W.) received a favourable opinion from the North of Scotland Research Ethics Committee (ref 08/S0802/84 approval date 12 February 2009). All subjects gave written informed consent and the study was conducted in accordance with the Declaration of Helsinki.

### 2.3. N-Glycome Analysis

Prior to any analytical procedure, each sample was allocated to the predefined position on 96-well plate, following a predetermined experimental design, which was blocked on case–control status, sex and age information. All plates included standard and blank samples for quality control and batch correction.

#### 2.3.1. IgG Isolation from Human Plasma

IgG was isolated by affinity chromatography using protein G monolithic plate (Bia Separations, Slovenia) as described previously [22]. In brief, plasma samples (70 μL) were diluted 7 × with phosphate-buffered saline (PBS, Merck, Germany) and transferred to 96-well protein G plate, then immediately washed with PBS. In the final step, IgG was eluted with 1 mL of 0.1 M formic acid (Merck, Germany) and instantly neutralized with 170 µL of 1 M ammonium bicarbonate (Across Organics, USA).

#### 2.3.2. N-Glycan Release from IgG and Total Plasma Proteins

Deglycosylation of IgG and plasma proteins was performed as described previously [22]. Aliquots of IgG eluates (300 μL) were dried in a vacuum centrifuge. After drying, IgG was denatured with the addition of 30 μL of 1.33% (*w/v*) sodium dodecyl sulphate (SDS) (Invitrogen, USA) and by 10 min incubation at 65 °C. Plasma samples (10 μL) were denatured with the addition of 20 μL of 2% (*w/v*) SDS and by 10 min incubation at 65 °C. From this point onwards N-glycan processing was identical for both IgG and plasma samples. After denaturation, 10 μL of 4% (*v/v*) Igepal-CA630 (Sigma Aldrich, USA) was added to the samples, followed by 1.2 U of PNGase F (Promega, USA). Samples were incubated overnight at 37 °C.

#### 2.3.3. Fluorescent Labelling and HILIC-SPE Purification of Released N-Glycans

N-glycans released from IgG and plasma proteins were labelled using 2-aminobenzamide (2-AB). To each sample 25 μL of labelling mixture was added consisting of 2-AB (19.2 mg/mL; Sigma Aldrich, USA) and 2-picoline borane (44.8 mg/mL; Sigma Aldrich, USA) in dimethyl sulfoxide (Sigma Aldrich, USA) and glacial acetic acid (Merck, Germany) mixture (70:30 *v/v*). Afterwards, samples were incubated for 2 h at 65 °C. Following the incubation, 700 μL of cold ACN (J.T. Baker, USA) was added to each sample, to obtain a final concentration of 96% (*v/v*) ACN. Samples were then applied to 0.2 μm GHP filer plate (Pall Corporation, USA), positioned on a vacuum manifold (Millipore Corporation, USA) used for solvent discharge. Samples were then washed 5x with 96% ACN and purified N-glycans were eluted from the filter plate using ultra-pure water. Eluates were stored at −20 °C.

#### 2.3.4. N-Glycan Profiling and Structure Assignment

**Figure 1:**
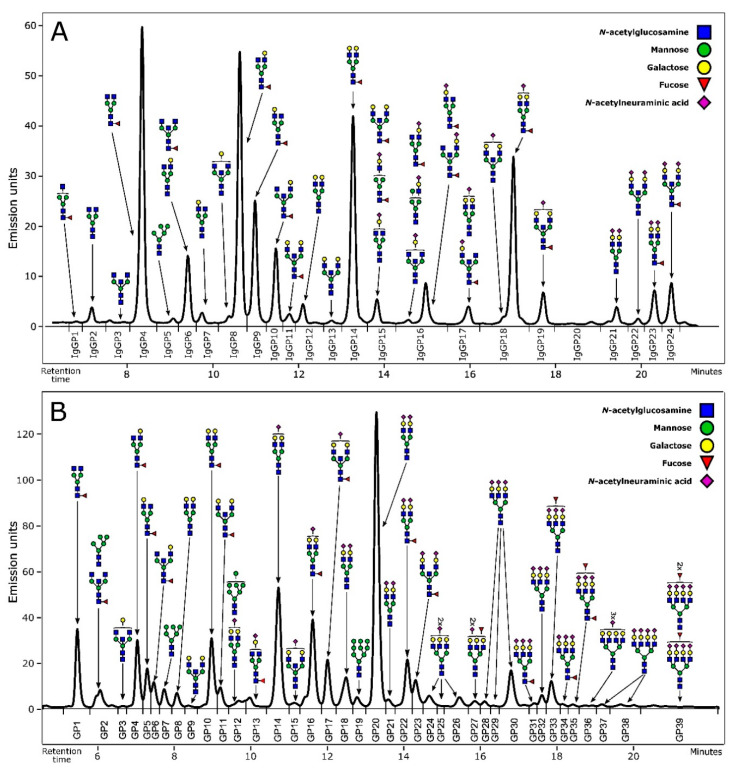
Representative chromatograms of HILIC-UPLC-FLR profiled N-glycans. (**a**) IgG N-glycome profile with graphical representation of the glycan structures corresponding to every IgG glycan peak (IgGP). (**b**) Plasma protein N-glycome profile with graphical representation of the most abundant structures corresponding to every plasma glycan peak (GP).

Fluorescently labelled N-glycans were separated by hydrophilic interaction liquid chromatography (HILIC) on Acquity ultra performance liquid chromatography (UPLC) H-Class instrument (Waters, USA). The instrument consisted of a quaternary solvent manager (QSM), a sample manager (SM) and a fluorescence (FLR) detector, and was controlled by Empower 3 software, build 3471 (Waters, USA). Excitation wavelength was set at 250 nm and emission wavelength at 428 nm. For N-glycan separation Waters BEH Glycan chromatography column was used. The calibration of the system was performed using an external standard containing hydrolysed and 2-AB labelled glucose oligomers, which were used for the conversion of individual glycans’ retention times to glucose units (GU). All chromatograms were separated in the same manner into 24 peaks for IgG N-glycans (IgGP1–IgGP24) and 39 peaks for plasma protein N-glycans (GP1–GP39) (Figure 1). Glycan peaks were then analysed based on their elution positions and measured in GU, and compared to the reference values in the “GlycoStore” database (available at https://glycostore.org/) for structure assignment. All glycan structures were further confirmed with MS/MS analysis by HILIC-UPLC coupled to a Compact ESI-QTOF-MS system via Ion Booster ion source (Bruker Daltonics, Germany). The instrument was under the control of Hystar software version 3.2 (Bruker Daltonics). The instrument was operated in a positive ion mode with capillary voltage set to 2250 V and nebulizing gas at pressure of 5.5 Bar. Drying gas (nitrogen) was applied to source at a flow rate of 4 L/min and temperature of 150 °C, while vaporizer temperature was set to 200 °C and flow rate of 5 L/min. Nitrogen was used as a source gas, while argon was used as a collision gas. Spectra were recorded in m/z range of 150–4000 at 0.5 Hz frequency. MS/MS analysis was performed using Auto MS/MS mode, which selects three precursors with the highest intensities for CID fragmentation. Glycan compositions and structural features were assigned using DataAnalysis, GlycoWorkbench and Glycomode software tools, based on obtained MS and MS/MS spectra. Detailed description of assigned glycan structures within each glycan peak is provided in Table S1. Specific glycosylation features, including sialylation, fucosylation, bisection, galactosylation and degree of glycan branching were described using derived traits, which are calculated from initial directly measured glycan traits. For IgG N-glycome, nine derived traits were calculated from 24 initial IgG glycan peaks (Table S2), while for plasma N-glycome, 16 derived traits were calculated using 39 initial plasma glycan peaks (Table S3).

### 2.4. Statistical Analysis

Relative glycan abundances (calculated as a percentage of the given glycan structure in the overall chromatogram) obtained by UPLC-FLR profiling were logit transformed to log-odds and then batch corrected using ComBat method (R package sva [23]). Derived glycan traits were calculated using batch corrected glycan data. Batch corrected data were used to estimate the difference in abundance of each glycan structure or derived glycan trait between subjects with MS and healthy controls by fitting a linear model with glycan log-odds as dependent variable and investigated group as independent variable (MS participants—cases and healthy controls), adjusted for sex and age. Pairwise comparisons between the groups for each glycome (IgG or plasma) were done using t-tests and presented as odds ratio (OR). False discovery rate (FDR) for all tests was controlled according to Benjamini–Hochberg’s procedure. Elastic net regularised logistic regression was used to estimate the power of glycans in multiple sclerosis classification with disease status as dependent variable, and sex and age (null model) or sex, age and all directly measured glycans (full model) as independent variables. Hyperparameters of the elastic net (α = 0.1, λ = 0.01) were estimated by minimizing 10-fold cross validated misclassification error (R package glmnet [24]). Results are presented with receiver operating characteristic (ROC) curves, whose areas under the curve (AUC) were compared using bootstrap test (R package pROC [25]). Statistical analysis was performed in the R programming language (version 3.5.2).

## 3. Results

### 3.1. Alterations of N-Glycome Composition in Multiple Sclerosis

We chromatographically profiled and quantified both IgG and plasma protein N-glycome in 83 MS cases and 88 controls. Based on directly measured glycans, nine IgG and 16 plasma derived glycan traits were calculated, representing composite traits that average particular glycan features, such as galactosylation, sialylation, fucosylation and the like.

Firstly, we wanted to examine differences in glycan abundances between multiple sclerosis subjects and the controls using a linear model. Comparison of multiple sclerosis subjects against their age-, sex- and ancestry-matched controls showed that four out of 24 directly measured IgG N-glycan traits were significantly different between the groups (Table 2). Only one significantly changed glycan was of oligomannose type, while all the others were complex N-glycans. The plasma N-glycome showed more extensive changes in multiple sclerosis, where nine out of 39 directly measured initial glycan traits differed significantly between the cases and the controls (Table 2). The majority of plasma glycans which demonstrated substantial changes in the disease were highly branched complex structures. The aforementioned prominent alterations in initial IgG and plasma glycan traits are also depicted in Figure S1 and Figure S2, respectively.

As our case group included subjects with four different disease courses, we wanted to examine whether glycans have a value in distinguishing particular types of MS, i.e., if they are able to stratify MS subjects alone. Here, we did not observe any statistically significant alterations in glycan abundances that could be associated with a specific MS type, either due to the limited sample size or generally similar effects of different MS types on the cellular level, which could then not be reflected in glycosylation alterations.

### 3.2. Complexity of Glycans Associated with Multiple Sclerosis

**Figure 2:**
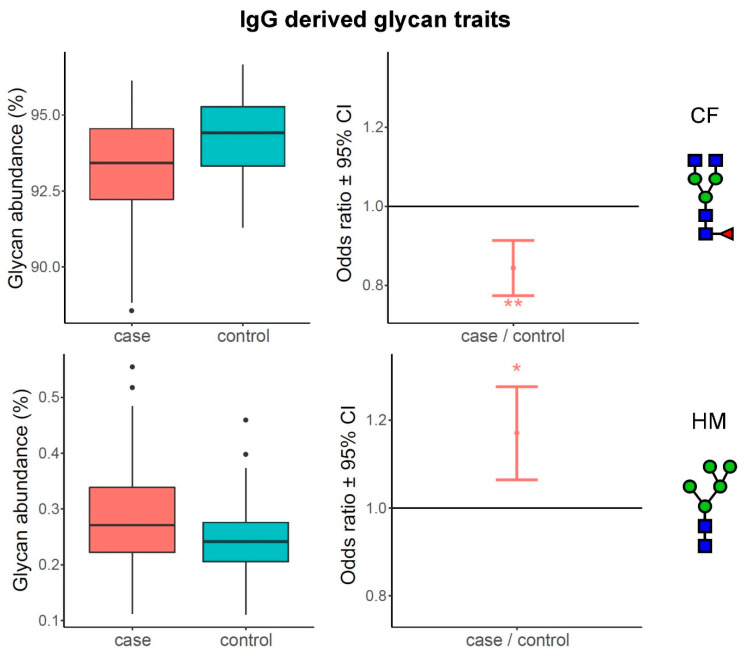
Most prominent differences between MS subjects and matched controls in IgG derived glycan traits. The left part of the figure shows boxplots representing differences in glycan abundances, while the right part shows odds ratio plots, resulting from post-hoc tests. Boxes in the boxplot are ranging from 25th to 75th percentile, with the median represented as a line inside the box. The whiskers of the boxplot extend from both percentiles, 75th percentile for upper whisker and 25th for lower whisker, to the values within 1.5 × IQR. IQR is the inter-quartile range, also known as the distance from the first to the third quartile. Data outside the whiskers’ ends are outliers, represented with black dots, and are plotted individually. On the odds ratio plots, middle dot represents odds ratio value for represented derived glycan trait surrounded by its 95% confidence interval (CI). Odds ratio of 1.0 means that there is no difference between compared groups for the given trait. Asterisk symbols represent statistical significance: * < 0.05; ** < 0.01.

**Figure 3:**
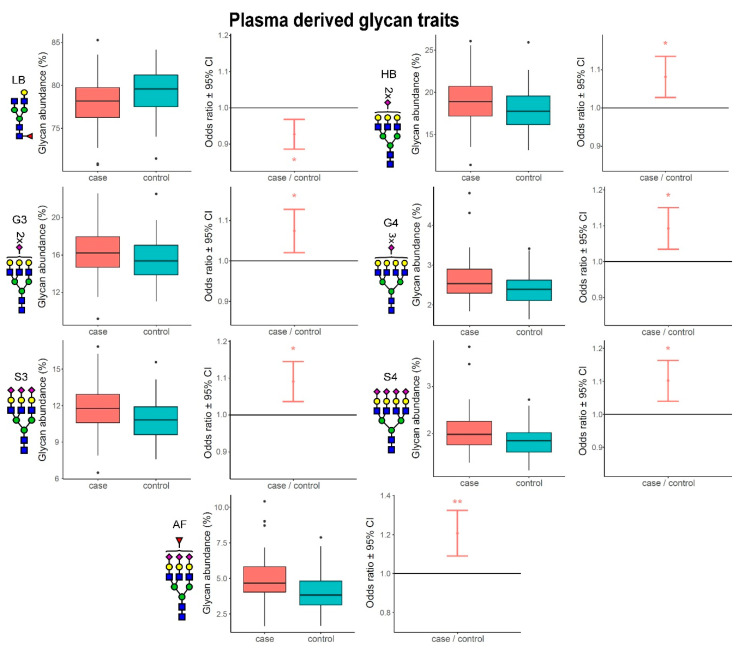
Most prominent differences between MS subjects and matched controls in plasma protein derived glycan traits. The left part of the figure shows boxplots representing differences in glycan abundances, while the right part shows odds ratio plots, resulting from post-hoc tests. Boxes in the boxplot are ranging from 25th to 75th percentile, with the median represented as a line inside the box. The whiskers of the boxplot extend from both percentiles, 75th percentile for upper whisker and 25th for lower whisker, to the values within 1.5 × IQR. IQR is the inter-quartile range, also known as the distance from the first to the third quartile. Data outside the whiskers’ ends are outliers, represented with black dots, and are plotted individually. On the odds ratio plots, middle dot represents odds ratio value for represented derived glycan trait surrounded by its 95% confidence interval (CI). Odds ratio of 1.0 means that there is no difference between compared groups for the given trait. Asterisk symbols represent statistical significance: * < 0.05; ** < 0.01.

Using linear models, we also tested the associations of composite derived glycan traits with multiple sclerosis, by comparing MS subjects to their matched controls. As previously mentioned, derived traits average particular glycosylation features and are thereby more closely related to the individual enzymatic activities. Out of nine calculated IgG N-glycosylation derived traits, two of them were significantly altered in multiple sclerosis—core fucosylation was significantly decreased (OR = 0.84, adjusted *p*-value = 6.96 × 10^−3^), while high mannose glycans were significantly increased (OR = 1.17, adjusted *p*-value = 1.48 × 10^−2^). For plasma N-glycome, seven derived traits out of 16 calculated were significantly different when compared between MS subjects and their matched controls. In particular, high-branched glycans (OR = 1.08, adjusted *p*-value = 2.19 × 10^−2^), antennary fucosylation (OR = 1.21, adjusted *p*-value = 6.78 × 10^−3^), trigalactosylation (OR = 1.07, adjusted *p*-value = 4.28 × 10^−2^), tetragalactosylation (OR = 1.09, adjusted *p*-value = 1.87 × 10^−2^), trisialylation (OR = 1.09, adjusted *p*-value = 1.66 × 10^−2^) and tetrasialylation (OR = 1.10, adjusted *p*-value = 1.66 × 10^−2^) were all significantly increased in the MS group (Table S4). Conversely, only low-branched (mono- and bi-antennary) glycans decreased in MS subjects (OR = 0.93, adjusted *p*-value 1.66 × 10^−2^). The most prominent differences in IgG and plasma protein derived glycan traits are depicted in Figure 2 and Figure 3, respectively.

Our results clearly show that all statistically significant changes in the plasma protein N-glycome, occurring in multiple sclerosis, are leading towards increased complexity of glycans, driven by elevation of high-branched, tri-and tetra-antennary structures bearing multiple galactose and sialic acid residues, and by concomitant decrease in low-branched, mono- and bi-antennary structures. This increase in glycan complexity cannot be observed for the IgG N-glycome, since the most complex glycan structures originating from IgG do not carry more than two antennae. However, it is interesting to observe that two types of fucosylation are the most prominently altered glycosylation traits and they are changing in the opposite directions. Namely, the core fucosylation of the IgG glycans decreases (as it does in plasma glycome, although non-significantly), while antennary fucosylation of plasma glycans increases, additionally contributing to their complexity.

### 3.3. Glycans Show Value in Disease Status Classification

Since N-glycans originating from IgG and total plasma proteins showed value in distinguishing MS cases from age- and sex-matched controls, we also wanted to examine their performance in the classification of disease status. Logistic regression was used, which incorporated all initial, directly measured N-glycans into the classification model, meaning that 24 glycan predictors were used to build an “IgG N-glycome classification model” and 39 were used to build a “plasma N-glycome classification model”. Their classification performance was tested by receiving operator characteristic (ROC) curve analysis. The resulting graphic visualization represents the performance of the N-glycome’s classification abilities by comparing glycan values in MS subjects against age- and sex-matched controls, expressed as area under the curve (AUC) values with their 95% confidence intervals. AUC value represents the probability that an individual has or does not have MS, based exclusively on his N-glycan abundances.

**Figure 4:**
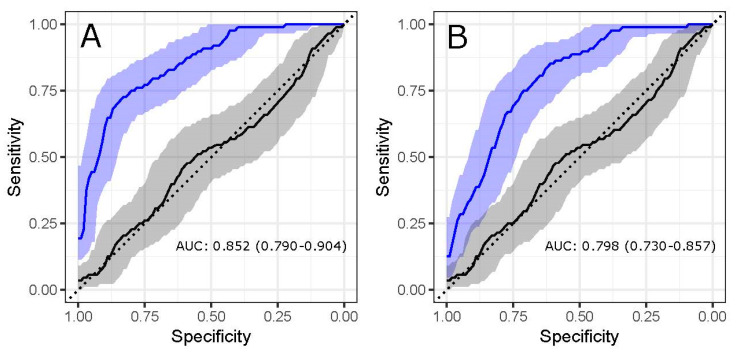
Classification performance of plasma protein and IgG N-glycome. ROC curves represent the performance of logistic regression model in classification of multiple sclerosis status using the total plasma protein N-glycome (**a**) and total IgG N-glycome (**b**). The blue line represents classification of disease status using the N-glycan model, while the black line represents performance of age and sex in disease status classification. Graphs are showing area under the curve (AUC) values with 95% confidence intervals.

Firstly, the discriminative performance of total plasma protein N-glycome was tested, resulting in an AUC of 0.852 (95% CI 0.790–0.904; *p* = 2.69 × 10^−11^), which indicated a strong discriminatory ability of total plasma N-glycans (Figure 4A). Detailed analysis revealed that plasma glycans GP14 (A2G2S1), GP5 (FA2[3]G1), GP25(A3G3S2) and GP6(FA2[6]BG1) contributed the most to the successful classification performance of the model (Figure S3).

Secondly, we examined the classification performance of the IgG N-glycome, which conferred a good discriminatory ability, with an AUC of 0.798 (95% CI 0.730–0.857, *p* = 1.1314 × 10^−7^) (Figure 4B); however, the plasma N-glycome again exhibited a higher potential as an MS biomarker. Analysis of the logistic regression model pinpointed IgG glycans IgGP21 (A2G2S2), IgGP9 (FA2[3]G1), IgGP7 (A2[3]G1) and IgGP11 (FA2[3]BG1) as the most informative structures in the classification model (Figure S3).

## 4. Discussion

To the best of our knowledge, this is the first study to compare both IgG and plasma protein N-glycosylation patterns between multiple sclerosis subjects and age- and sex-matched controls, while also examining glycan classification performance. We have observed extensive disease-related alterations in the N-glycome composition, predominantly originating from plasma glycoproteins, nonetheless, the IgG N-glycome exerted significant changes, as well.

The most prominent changes in the plasma protein N-glycome associated with MS can be summarized as significant increase in high-branched structures, bearing multiple galactose and sialic acid residues, meaning that MS is marked by increased complexity of the plasma N-glycome. Consequently, low-branched glycans have a lower abundance in MS cases than in matched controls. This increase in plasma N-glycome complexity is consistent with previous studies that have examined glycosylation changes in Alzheimer’s disease (AD) [26,27]. Nonetheless, AD studies have also reported increased bisection of plasma N-glycans, as well as reduction of core fucosylated digalactosylated biantennary glycans [26,27], while we observed MS-related increase in antennary fucosylation. Additionally, a recent study analysing plasma N-glycome alterations in Parkinson’s disease (PD) reported lower levels of tri- and tetra-antennary glycans containing two or three sialic acid residues [28], opposed to the increased complexity of plasma N-glycome observed in MS and AD. Combined, the aforementioned findings indicate that each one of CNS-affecting diseases has a unique combination of relevant plasma glycosylation changes. As already mentioned, herein we observed an increase in antennary fucosylation of total plasma glycoproteins. Antennary fucosylated, sialylated structures build sialyl-Lewis X structures on acute phase proteins, whose concentration increases in inflammation [29]. Sialyl-Lewis X structures are also recognized by selectins and involved in essential immune processes, such as leukocyte rolling, adhesion and trafficking [30]. Various adhesion molecules play a crucial role in the MS pathogenesis [31], as they enable binding of circulating autoreactive immune cells to the CNS endothelial cells and migration through the blood-brain barrier—an essential step in the initiation of CNS inflammation [32]. Activation of endothelial cells and immune cells in blood results in rapid upregulation and possible shedding of adhesion molecules in serum and CSF [33], potentially altering composition of the plasma N-glycome. The observed plasma protein glycosylation alterations could be attributed to either variation in glycan occupancy on certain proteins or to variation in protein concentration. At this point, we cannot determine their exact source; however, plasma N-glycome likely reflects an overall trend of glycosylation changes, mirroring both alterations in glycosylation of individual proteins as well as changes in their concentration.

Disrupted immune self-recognition is a hallmark of autoimmune disorders, including MS, resulting in pathological immune responses that target either cellular or organ-specific self-antigens [34], leading to systemic inflammation. Consequently, the produced IgG autoantibodies are involved in tissue damage [35]. Our results demonstrate marked changes in IgG N-glycome composition in MS subjects, specifically the significantly altered abundance of high mannose glycans and core fucosylation. Core fucosylation of IgG Fc fragment is considered to be a safety switch for ADCC, since it sterically disrupts binding of the Fc region to the FcγRIIIa receptor on effector cells [14]. Conversely, decrease or loss of core fucose increases the proinflammatory potential of IgG and results in overactivated ADCC, known to have a major role in the pathogenesis of various autoimmune disorders. The MS patients participating in our study had substantially lowered IgG core fucosylation, implying the inefficient control and overactivation of ADCC, which would additionally contribute to the MS pathogenesis. Other prominent change of the IgG N-glycome was an increase of high mannose structures in MS subjects, which has not been reported previously. The high mannose glycans are usually present in high abundance on various pathogens, which are then recognized and bound by mannose-binding lectin (MBL), thereby initiating the lectin pathway of complement activation. In disease, MBL contributes to the inflammation and has already been implicated in the pathogenesis of rheumatoid arthritis, as it was shown that multiple presentation of agalactosylated IgG glycoforms to MBL results in activation of the complement [36]. Significantly elevated levels of MBL were reported [37] in MS as well, while a recent study [38] demonstrated that complement activation occurs in the MS cortical grey matter lesions. Elevated levels of high mannose IgG glycoforms increase the chance of complement activation through MBL recognition, which would contribute to the demyelination and irreversible progression of MS. To summarize, both significant alterations in IgG N-glycome composition are supporting inflammatory processes in MS; however, additional studies are needed to determine the pathways involved.

Apart from exploring the differences in IgG and plasma protein N-glycomes between MS cases and healthy controls, we also wanted to investigate if glycans show value in differentiating various MS subtypes. Unfortunately, no significant differences in glycosylation were observed between subjects with RRMS, PPMS, SPMS with relapses and SPMS without relapses, possibly due to limited number of participants with different MS subtypes or due to the general similarity of pathological changes at the cellular level.

The scope of previous research on glycosylation alterations in MS is rather modest. One study [20], exploring IgG1 Fc-glycome alterations in MS, did not observe significant glycosylation changes in serum-derived IgG1, but only in its CSF fraction, possibly due to a smaller sample size or the fact that the authors monitored Fc glycosylation only (herein the pool of Fc and Fab IgG glycans was profiled). The same study demonstrated that IgG1 glycosylation patterns differ between CSF and serum, even in controls without intrathecal IgG synthesis, which hinders the clinical utility of these comparisons. However, the authors observed prominent MS-related glycosylation alterations in CSF-derived IgG1, where increased bisecting GlcNAc, increased core fucosylation and decreased galactosylation were observed [20]. Elevated bisecting GlcNAc and reduced galactosylation are known to enhance IgG effector functions; however elevated core fucosylation has the opposite effect. Nonetheless, another recent study conversely observed that IgG from CSF of MS patients is characterized by significantly reduced bisecting GlcNAc [19], confounding the previous observations. Conversely, all glycosylation changes observed herein are jointly suggesting the enhanced proinflammatory potential of the circulating IgG.

When addressing the possibility of disease status prediction using solely N-glycans, we demonstrated that the N-glycome offers quite promising prospects. The plasma N-glycome was able to successfully distinguish individuals with MS from healthy controls, showing an AUC of 0.852, while IgG N-glycome performed modestly, with an AUC of 0.798. Considerable effort has been invested into search of predictive MS biomarkers, as they could aid in identification of individuals at high risk of developing MS. For instance, a recent study [39] investigated markers in CSF (TNF-α, IL-10, CXCL13, and NF-L) which may predict a diagnosis of MS in patients with acute optic neuritis (ON), an early inflammatory event. The tested multivariable prediction model, which included the aforementioned CSF markers, resulted in an AUC of 0.89 (95% CI 0.77–1), which is comparable to our plasma N-glycome model. Altogether, our results show that the N-glycome exhibits a considerable potential for predicting MS status in an individual. However, we need to acknowledge the possibility that at least some of the observed glycome changes are secondary to the disease, warranting further research that would include individuals at high MS risk, such as presence of early inflammatory events.

## 5. Conclusions

In conclusion, our study enriches current findings on proinflammatory glycosylation profile of IgG in multiple sclerosis, but also provides new insights into plasma N-glycosylation alterations, suggesting that glycans might have a role in MS aetiology that should be further explored. Glycans also show good classification ability, as they were able to distinguish MS patients from their controls with a satisfactory performance. Our study resulted in one interesting observation—core fucosylation of IgG and antennary fucosylation of plasma glycoproteins show opposite directions of effect in MS, demonstrating how the same monosaccharide residue can have an impact on diverse immune processes. It would be worth exploring whether these alterations have an influence on the disease course and outcomes, since, based on the existing knowledge, they definitively contribute to the inflammation and tissue damaging processes. Monitoring glycosylation changes has untapped potential in the biomarker field; however, additional studies with a larger sample sizes are warranted.

## Figures and Tables

**Table 1:** Clinical characteristics of the study participants. ^1^

	Case	Control	*p*-Value
Participants, N	83	88	
Sex, N_female_ (%)	58 (70)	62 (70)	1.000
Age, median (min-max)	52 (29–77)	50 (28–77)	0.830
Type of multiple sclerosis, N (%)			
Relapsing remitting	31 (37)		
Secondary progressive with relapses	25 (30)		
Secondary progressive without relapses	15 (18)		
Primary progressive	12 (14)		
Expanded Disability Status Scale, median (IQR)			
Relapsing remitting	2.0 (1.0–4.5)		
Secondary progressive with relapses	6.0 (5.0–8.0)		
Secondary progressive without relapses	6.5 (6.0–7.5)		
Primary progressive	6.0 (6.0–7.5)		

^1^ Difference in sex was tested using Fisher-exact test, while difference in age was tested using Wilcoxon rank-sum test. IQR—interquartile range.

**Table 2:** Comparison of IgG and plasma protein initial glycan traits between subjects with MS and matched controls. ^1^

Sample	Glycan Peak	Glycan Structure	Odds Ratio	95% CI	*p*-Value	Adjusted *p*-Value
IgG	IgGP5	M5	1.17	1.06–1.28	7.48 × 10^−4^	1.48 × 10^−2^
IgG	IgGP9	FA2[3]G1	0.92	0.88–0.96	6.38 × 10^−4^	1.48 × 10^−2^
IgG	IgGP17	FA2[3]BG1S1, A2G2S1	1.16	1.05–1.27	1.95 × 10^−3^	3.21 × 10^−2^
IgG	IgGP21	A2G2S2	1.24	1.09–1.38	3.78 × 10^−4^	1.48 × 10^−2^
**Sample**	**Glycan Peak**	**Glycan Structure**	**Odds Ratio**	**95% CI**	***p*-Value**	**Adjusted *p*-Value**
plasma	GP4	FA2[6]G1	0.90	0.84–0.97	4.19 × 10^−3^	3.57 × 10^−2^
plasma	GP5	FA2[3]G1	0.86	0.79–0.94	1.37 × 10^−3^	1.87 × 10^−2^
plasma	GP10	FA2G2	0.88	0.82–0.95	1.04 × 10^−3^	1.86 × 10^−2^
plasma	GP27	A3F1G3S2	1.18	1.06–1.31	1.67 × 10^−3^	1.93 × 10^−2^
plasma	GP32	A3G3S3	1.13	1.04–1.21	1.42 × 10^−3^	1.87 × 10^−2^
plasma	GP33	A3F1G3S3	1.20	1.08–1.33	6.12 × 10^−4^	1.66 × 10^−2^
plasma	GP35	FA3F1G3S3, A4F1G4S3	1.23	1.11–1.34	1.94 × 10^−5^	1.57 × 10^−3^
plasma	GP38	A4G4S4, A4F1G4S4	1.08	1.02–1.14	4.14 × 10^−3^	3.57 × 10^−2^
plasma	GP39	A4F1G4S4, A4F2G4S4	1.18	1.08–1.27	1.50 × 10^−4^	6.78 × 10^−3^

^1^ Differences are expressed as odds ratio values, resulting from post-hoc tests. Glycan data were adjusted for age and sex, while false discovery rate was controlled using Benjamini–Hochberg method. Only statistically significant results are shown.

## Supplementary Materials

The following are available online at https://www.mdpi.com/2227-9059/8/10/410/s1, Table S1: Compositions and structural features of N-glycans corresponding to every individual plasma (GP) and IgG (IgGP) glycan peak, Table S2: IgG derived glycan traits calculated out of 24 directly measured IgG glycan peaks, Table S3: Plasma derived glycan traits calculated out of 39 directly measured plasma protein glycan peaks, Figure S1: Most prominent differences between MS subjects and matched controls in directly measured IgG glycans, Figure S2: Most prominent differences between MS subjects and matched controls in directly measured plasma protein glycans, Table S4: Comparison of IgG and plasma protein derived glycan traits between subjects with MS and matched controls, Figure S3: Contribution of individual glycans in multiple sclerosis classification model.
