# Supplementary material for: Glycosylation Alterations in Multiple Sclerosis Show Increased Proinflammatory Potential
Source: Biomedicines. 2020 Oct 13;8(10):410. doi: 10.3390/biomedicines8100410 (PMC7599553; doi:10.3390/biomedicines8100410)
Supplement: Supplementary file 1 [file biomedicines-08-00410-s001.pdf]

Supplementary Materials

Glycosylation alterations in multiple sclerosis show increased proinflammatory potential

Ana Cvetko [1], Domagoj Kifer [1], Olga Gornik [1,4], Lucija Klarić [2], Elizabeth Visser [3], Gordan Lauc [1,4,*], James F. Wilson [2,5] and Tamara Štambuk [1,4,*]

[1] Faculty of Pharmacy and Biochemistry, University of Zagreb, 10000 Zagreb, Croatia; acvetko@pharma.hr (A.C.), dkifer@pharma.hr (D.K.), ogornik@pharma.hr (O.G.)

[2] MRC Human Genetics Unit, Institute of Genetics and Molecular Medicine, University of Edinburgh, Edinburgh EH4 2XU, United Kingdom; klaric.lucija.klaric@gmail.com (L.K.), Jim.Wilson@ed.ac.uk (J.F.W.)

[3] The Institute of Applied Health Sciences, MRC University of Aberdeen, Aberdeen AB25 2ZD, United Kingdom; e.visser@abdn.ac.uk (E.V)

[4] Genos Glycoscience Research Laboratory, 10000 Zagreb, Croatia

[5] Centre for Global Health Research, Usher Institute, University of Edinburgh, Edinburgh EH8 9AG, United Kingdom

[*] Correspondence: tstambuk@genos.hr (T.Š.); glauc@pharma.hr (G.L.); Tel.: +385 1 6394 467 (G.L.)

Table S1 Compositions and structural features of N-glycans corresponding to every individual plasma (GP) and IgG (IgGP) glycan peak.

Plasma glycan peak	*Observed m/z [M+H]+*	*Observed m/z [M+2H]2+*	*Glycan composition**	*Glycan structure#*	*Description*	*Formula*
GP1	1563.5862	-	H3N4F1	FA2	core fucosylated, biantennary	GP1 / GP * 100
GP2	1786.6643;<br>1355.4825	-<br>-	H3N5F1;<br>H5N2	FA2B;<br>M5	core fucosylated, biantennary with bisecting GlcNAc;<br>high mannose	GP2 / GP * 100
GP3	1802.6593	-	H4N5	A2BG1	monogalactosylated, biantennary with bisecting GlcNAc	GP3 / GP * 100
GP4	1745.6365	-	H4N4F1	FA2[6]G1	core fucosylated and monogalactosylated, biantennary	GP4 / GP * 100
GP5	1745.6366	-	H4N4F1	FA2[3]G1	core fucosylated and monogalactosylated, biantennary	GP5 / GP * 100
GP6	1948.7116	974.8585	H4N5F1	FA2[6]BG1	core fucosylated and monogalactosylated, biantennary with bisecting GlcNAc	GP6 / GP * 100
GP7	1517.5309;<br>1948.7125	-<br>-	H6N2;<br>H4N5F1	M6;<br>FA2[3]BG1	high mannose;<br>core fucosylated and monogalactosylated, biantennary with bisecting GlcNAc	GP7 / GP * 100
GP8	1761.6325	-	H5N4	A2G2	digalactosylated, biantennary	GP8 / GP * 100
GP9	1964.7084	-	H5N5	A2BG2	digalactosylated, biantennary with bisecting GlcNAc	GP9 / GP * 100
GP10	1907.6891	-	H5N4F1	FA2G2	core fucosylated, digalactosylated, biantennary	GP10 / GP * 100
GP11	2110.7604	1055.8841	H5N5F	FA2BG2	core fucosylated, digalactosylated, biantennary with bisecting GlcNAc	GP11 / GP * 100
GP12	1679.5653;<br>2052.7202;<br>1849.6453;<br>2093.7602	840.2521;<br><br>925.8160;	H7N2;<br>H5N4S1;<br>H5N3S1;<br>H4N5S1	M7;<br>A2G2S1;<br>A1M4G1S1;<br>A2BG1S1	high mannose;<br>digalactosylated, monosialylated, biantennary;<br>hybrid, monogalactosylated, monosialylated, monoantennary;	GP12 / GP * 100

					monogalactosylated, monosialylated, biantennary with bisecting GlcNAc	
GP13	2036.7265;<br>2239.8003	-<br>-	H4N4F1S1;<br>H4N5F1S1	FA2G1S1;<br>FA2BG1S1	core fucosylated, monogalactosylated and monosialylated biantennary;<br>core fucosylated, monogalactosylated and monosialylated biantennary with bisecting GlcNAc	GP13 / GP * 100
GP14	2052.7207	-	H5N4S1	A2G2S1	digalactosylated and monosialylated biantennary	GP14 / GP * 100
GP15	2255.7954	-	H5N5S1	A2BG2S1	digalactosylated and monosialylated biantennary with bisecting GlcNAc	GP15 / GP * 100
GP16	2198.7771	1099.8897	H5N4F1S1	FA2G2S1	core fucosylated, digalactosylated and monosialylated biantennary	GP16 / GP * 100
GP17	2401.8537	1201.4297	H5N5F1S1	FA2BG2S1	core fucosylated, digalactosylated and monosialylated biantennary with bisecting GlcNAc	GP17/ GP * 100
GP18	2343.8090;<br>2489.8631	1172.4072;<br>1245.4346	H5N4S2;<br>H5N4F1S2	A2G2S2;<br>FA2G2S2	digalactosylated and disialylated biantennary;<br>core fucosylated, digalactosylated and disialylated biantennary	GP18 / GP * 100
GP19	2003.6778	-	H9N2	M9	high mannose	GP19 / GP * 100
GP20	2343.8084	1172.4068	H5N4S2	A2G2S2	digalactosylated and disialylated biantennary	GP20 / GP * 100
GP21	2343.7938;<br>2417.8385;<br>2489.8675;<br>2546.8731;<br>2563.9034	1172.3986;<br>1209.4216;<br>1245.4324;<br>1273.9417;<br>1282.4526	H5N4S2;<br>H6N5S1;<br>H5N4F1S2;<br>H5N5S2;<br>H6N5F1S1	A2G2S2;<br>A3G3S1;<br>FA2G2S2;<br>A2BG2S2;<br>A3F1G3S1	digalactosylated and disialylated biantennary;<br>trigalactosylated and monosialylated triantennary;<br>core fucosylated, digalactosylated and disialylated biantennary;<br>digalactosylated and disialylated biantennary with bisecting GlcNAc;<br>antennary fucosylated, trigalactosylated and monosialylated triantennary	GP21 / GP * 100
GP22	2489.8653	1245.4354	H5N4F1S2	FA2G2S2	core fucosylated, digalactosylated and disialylated biantennary	GP22 / GP * 100

GP23	2692.9402	1346.9743	H5N5F1S2	FA2BG2S2	core fucosylated, digalactosylated and disialylated biantennary with bisecting GlcNAc	GP23 / GP * 100
GP24	2708.9912; 2563.8988	1354.9717; 1282.4540	H6N5S2; H6N5F1S1	A3G3S2; A3F1G3S1	trigalactosylated and disialylated triantennary; antennary fucosylated, trigalactosylated and monosialylated triantennary	GP24 / GP * 100
GP25	2708.9367; 2854.9980	1354.9713; 1427.9960	H6N5S2; H6N5F1S2	A3G3S2; A3F1G3S2	trigalactosylated and disialylated triantennary; antennary fucosylated, trigalactosylated and disialylated triantennary	GP25 / GP * 100
GP26	2708.9379; 2854.9924	1354.9705 1427.9961	H6N5S2; H6N5F1S2	A3G3S2; FA3G3S2	trigalactosylated and disialylated triantennary; core fucosylated, trigalactosylated and disialylated triantennary	GP26 / GP * 100
GP27	2854.9916	1427.9980	H6N5F1S2	A3F1G3S2	antennary fucosylated, trigalactosylated and disialylated triantennary	GP27 / GP * 100
GP28	3000.0218; 2854.9941	1500.5163; 1428.0000	H6N5S3; H6N5F1S2	A3G3S3; A3F1G3S2	trigalactosylated and trisialylated triantennary; antennary fucosylated, trigalactosylated and disialylated triantennary	GP28 / GP * 100
GP29	3000.0268 2854.9869	1500.5155; 1428.0008	H6N5S3; H6N5F1S2	A3G3S3; A3F1G3S2	trigalactosylated and trisialylated triantennary; antennary fucosylated, trigalactosylated and disialylated triantennary	GP29 / GP * 100
GP30	3000.0330	1500.5178	H6N5S3	A3G3S3	trigalactosylated and trisialylated triantennary	GP30 / GP * 100
GP31	3146.0844; 3000.0330	1573.5457; 1500.5189	H6N5F1S3; H6N5S3	FA3G3S3; A3G3S3	core fucosylated, trigalactosylated and trisialylated triantennary; trigalactosylated and trisialylated triantennary	GP31 / GP * 100
GP32	3000.0323	1500.5171	H6N5S3	A3G3S3	trigalactosylated and trisialylated triantennary	GP32 / GP * 100
GP33	3146.0864	1573.5464	H6N5F1S3	A3F1G3S3	antennary fucosylated, trigalactosylated and trisialylated triantennary	GP33 / GP * 100
GP34	3146.0879; 3365.1553	1573.5463; 1683.0832	H6N5F1S3; H7N6S3	FA3G3S3; A4G4S3	core fucosylated, trigalactosylated and trisialylated triantennary; tetragalactosylated and trisialylated tetraantennary	GP34 / GP * 100

GP35	3292.1600; 3511.2335	1646.5752; 1756.1192	H6N5F2S3; H7N6F1S3	FA3F1G3S3; A4F1G4S3	core fucosylated, antennary fucosylated, trigalactosylated and trisialylated triantennary; antennary fucosylated, tetragalactosylated and trisialylated tetraantennary	GP35 / GP * 100
GP36	3365.1775; 3511.2223	1683.0865; 1756.1162	H7N6S3; H7N6F1S3	A4G4S3; A4F1G4S3	tetragalactosylated and trisialylated tetraantennary; antennary fucosylated, tetragalactosylated and trisialylated tetraantennary	GP36 / GP * 100
GP37	3656.2734; 3511.2223	1828.6331; 1756.1321	H7N6S4; H7N6F1S3	A4G4S4; A4F1G4S3	tetragalactosylated and tetrasialylated tetraantennary; antennary fucosylated, tetragalactosylated and trisialylated tetraantennary	GP37 / GP * 100
GP38	3656.5766; 3802.4774	1828.6420; 1901.6858	H7N6S4; H7N6F1S4	A4G4S4; A4F1G4S4	tetragalactosylated and tetrasialylated tetraantennary; antennary fucosylated, tetragalactosylated and tetrasialylated tetraantennary	GP38 / GP * 100
GP39	3802.4178; 3949.3420	1901.6858; 1974.6839	H7N6F1S4; H7N6F2S4	A4F1G4S4; A4F2G4S4	antennary fucosylated, tetragalactosylated and tetrasialylated tetraantennary; antennary difucosylated, tetragalactosylated and tetrasialylated tetraantennary;	GP39 / GP * 100
IgG glycan peak	***Observed m/z, $[M+H]^+$***	***Observed m/z, $[M+2H]^{2+}$***	***Glycan composition****	***Glycan structure***[#]	***Description***	***Formula***
IgGP1	1380.5101	-	H3N3F1	FA1	core fucosylated, monoantennary	IgGP1 / IgGP * 100
IgGP2	1437.5302	-	H3N4	A2	agalactosylated, biantennary	IgGP2 / IgGP * 100
IgGP3	1640.6062	-	H3N5	A2B	biantennary with bisecting GlcNAc	IgGP3 / IgGP * 100
IgGP4	1583.5818	-	H3N4F1	FA2	core fucosylated, biantennary	IgGP4 / IgGP * 100

IgGP5	1355.4761	-	H5N2	M5	high mannose	IgGP5 / IgGP * 100
IgGP6	1786.6998;<br>1599.5745	-	H3N5F1;<br>H4N4	FA2B;<br>A2[6]G1	core fucosylated, biantennary with bisecting GlcNAc;<br>monogalactosylated, biantennary	IgGP6 / IgGP * 100
IgGP7	1599.5766	-	H4N4	A2[3]G1	monogalactosylated, biantennary	IgGP7 / IgGP * 100
IgGP8	1802.6514<br>1745.6307	-<br>-	H4N5<br>H4N4F1	A2BG1;<br>FA2[6]G1	monogalactosylated, biantennary with bisecting GlcNAc;<br>core fucosylated and monogalactosylated, biantennary	IgGP8 / IgGP * 100
IgGP9	1745.6276	-	H4N4F1	FA2[3]G1	core fucosylated and monogalactosylated, biantennary	IgGP9 / IgGP * 100
IgGP10	1948.7034	974.8533	H4N5F1	FA2[6]BG1	core fucosylated and monogalactosylated, biantennary with bisecting GlcNAc	IgGP10 / IgGP * 100
IgGP11	1948.7022	-	H4N5F1	FA2[3]BG1	core fucosylated and monogalactosylated, biantennary with bisecting GlcNAc	IgGP11 / IgGP * 100
IgGP12	1761.6235	-	H5N4	A2G2	digalactosylated, biantennary	IgGP12 / IgGP * 100
IgGP13	1964.6982	-	H5N5	A2BG2	digalactosylated, biantennary with bisecting GlcNAc	IgGP13 / IgGP * 100
IgGP14	1907.6764	-	H5N4F1	FA2G2	core fucosylated, digalactosylated, biantennary	IgGP14 / IgGP * 100
IgGP15	1890.6524;<br>1833.6432;<br>2110.7544	-<br>-<br>1055.8796	H4N4S1;<br>H4N3F1S1<br>H5N5F1	A2G1S1;<br>FA1G1S1;<br>FA2BG2	monogalactosylated and monosialylated biantennary; core fucosylated, monogalactosylated and monosialylated monoantennary; core fucosylated, digalactosylated, biantennary with bisecting GlcNAc	IgGP15 / IgGP * 100
IgGP16	2093.7000<br>2036.7205;<br>1849.6587;	-<br>-<br>-	H4N5S1;<br>H4N4F1S1;<br>H5N3S1;	A2BG1S1;<br>FA2[6]G1S1;<br>A1M4G1S1;	monogalactosylated and monosialylated biantennary with bisecting GlcNAc;	IgGP16 / IgGP * 100

	2239.7949;<br>2036.7205;	-<br>-	H4N5F1S1;<br>H4N4F1S1	FA2[6]BG1S1;<br>FA2[3]G1S1	core fucosylated, monogalactosylated and monosialylated biantennary;<br>hybrid, monogalactosylated and monosialylated biantennary;<br>core fucosylated, monogalactosylated, monosialylated biantennary with bisecting GlcNAc;<br>core fucosylated, monogalactosylated and monosialylated biantennary	
IgGP17	2239.7899;<br>2052.7108	-<br>-	H4N5F1S1;<br>H5N4S1	FA2[3]BG1S1;<br>A2G2S1	core fucosylated, monogalactosylated, monosialylated biantennary with bisecting GlcNAc;<br>digalactosylated and monosialylated biantennary	IgGP17/ IgGP * 100
IgGP18	2255.7695;<br>2198.8215	-<br>-	H5N5S1;<br>H5N4F1S1	A2BG2S1;<br>FA2G2S1	digalactosylated and monosialylated biantennary with bisecting GlcNAc;<br>core fucosylated, digalactosylated and monosialylated biantennary	IgGP18 / IgGP * 100
IgGP19	2401.8390	1201.4220	H5N5F1S1	FA2BG2S1	core fucosylated, digalactosylated and monosialylated biantennary with bisecting GlcNAc	IgGP19 / IgGP * 100
IgGP20				structure not determined		IgGP20 / IgGP * 100
IgGP21	2343.8028	1172.4019	H5N4S2	A2G2S2	digalactosylated and disialylated biantennary	IgGP21 / IgGP * 100
IgGP22	2546.8809	1273.9423	H5N5S2	A2BG2S2	digalactosylated and disialylated biantennary with bisecting GlcNAc	IgGP22 / IgGP * 100
IgGP23	2489.8596	1245.4309	H5N4F1S2	FA2G2S2	core fucosylated, digalactosylated and disialylated biantennary	IgGP23 / IgGP * 100
IgGP24	2692.9315	1346.9673	H5N5F1S2	FA2BG2S2	core fucosylated, digalactosylated and disialylated biantennary with bisecting GlcNAc	IgGP24 / IgGP * 100

*composition abbreviations – Hx, number (x) of hexose residues; Nx, number (x) of GlcNAc residues; Fx, number (x) of fucose residues; Sx, number (x) of N-acetylneuraminic acid residues.

#structure abbreviations – all N-glycans have two core GlcNAcs; F at the start of the abbreviation indicates a core-fucose α1,6-linked to the inner GlcNAc; Mx, number (x) of mannose on core GlcNAcs; Ax, number of antenna (GlcNAc) on trimannosyl core; A2, biantennary with both GlcNAcs as β1,2-linked; A3, triantennary with a GlcNAc linked β1,2 to both mannose and the third GlcNAc linked β1,4 to the α1,3 linked mannose; A4, GlcNAcs linked as A3 with additional GlcNAc β1,6 linked to α1,6 mannose; B, bisecting GlcNAc linked β1,4 to β1,3 mannose; G(x), number (x) of β1,4 linked galactose on antenna; F(x), number (x) of fucose linked α1,3 to antenna GlcNAc; S(x), number (x) of sialic acids linked to galactoses.

Table S2 IgG derived glycan traits calculated out of 24 directly measured IgG glycan peaks.

Structural feature	***Formula***
Agalactosylation (G0)	IgGP1+IgGP2+IgGP4+IgGP6
Monogalactosylation (G1)	IgGP7+IgGP8+IgGP9+IgGP10+IgGP11+IgGP16
Digalactosylation (G2)	IgGP12+IgGP13+IgGP14+IgGP15+IgGP17+IgGP18+IgGP19+IgGP21+IgGP22+IgGP23+IgGP24
Neutral glycans (S0)	IgGP7+IgGP8+IgGP9+IgGP10+IgGP11+IgGP12+IgGP13+IgGP14+IgGP15
Monosialylation (S1)	IgGP16+IgGP17+IgGP18+IgGP19
Disialylation (S2)	IgGP21+IgGP22+IgGP23+IgGP24
Incidence of bisecting GlcNAc (B)	IgGP6+IgGP10+IgGP11+IgGP13+IgGP15+IgGP19+IgGP22+IgGP24
Core fucosylation (CF)	IgGP1+IgGP4+IgGP6+IgGP8+IgGP9+IgGP10+IgGP11+IgGP14+IgGP15+IgGP16+IgGP18+IgGP19+IgGP23+IgGP24
High mannose glycans (HM)	IgGP5

Table S3 Plasma derived glycan traits calculated out of 39 directly measured plasma protein glycan peaks.

Structural feature	***Formula***
Low branching (mono- and biantennary glycans) (LB)	GP1+GP2+GP3+GP4+GP5+GP6+GP8+GP9+GP10+GP11+0.5xGP12+GP13+GP14+ GP15+GP16+GP17+GP18+GP20+GP21+GP22+GP23
High branching (tri- and tetraantennary glycans) (HB)	GP24+GP25+GP26+GP27+GP28+GP29+GP30+GP31+GP32+GP33+GP34+ GP35+GP36+GP37+GP38+GP39
Agalactosylation (G0)	GP1+GP2
Monogalactosylation (G1)	GP3+GP4+GP5+GP6+GP13
Digalactosylation (G2)	GP8+GP9+GP10+GP11+0.5xGP12+GP14+GP15+GP16+GP17+GP18+GP20+GP21+GP22+GP23
Trigalactosylation (G3)	GP24+GP25+GP26+GP27+GP28+GP29+GP30+GP31+GP32+GP33+GP34+ GP35
Tetragalactosylation (G4)	GP36+GP37+GP38+GP39
Neutral glycans (S0)	GP1+GP2+GP3+GP4+GP5+GP6+GP8+GP9+GP10+GP11
Monosialylation (S1)	0.5xGP12+GP13+GP14+GP15+GP16+GP17
Disialylation (S2)	GP18+GP20+GP21+GP22+GP23+GP24+GP25+GP26+GP27
Trisialylation (S3)	GP28+GP29+GP30+GP31+GP32+GP33+GP34+GP35+GP36
Tetrasialylation (S4)	GP37+GP38+GP39
Incidence of bisecting GlcNAc (B)	GP2+GP3+GP6+GP9+GP11+GP15+GP17+GP23
Antennary fucosylation (AF)	GP27+GP33+GP35+GP39
Core fucosylation (CF)	GP1+GP2+GP4+GP5+GP6+GP10+GP11+GP13+GP16+GP17+GP22+GP23+GP31+GP34+GP35
High mannose structures (HM)	GP7+0.5xGP12+GP19

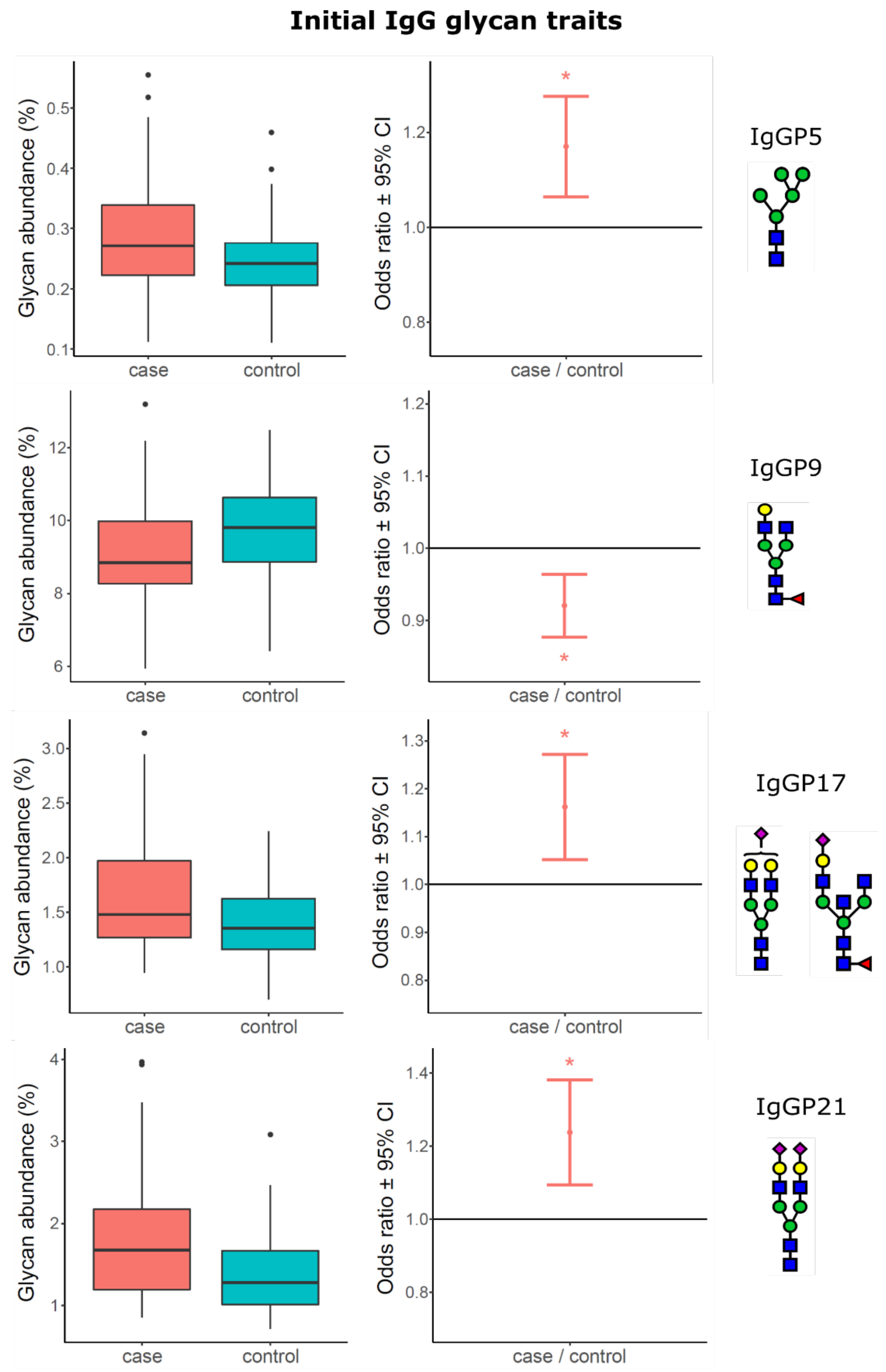

Figure S1 Most prominent differences between MS subjects and matched controls in directly measured IgG glycans. Left part of the figure shows boxplots representing differences in glycan abundances, while right part shows odds ratio plots, resulting from post-hoc tests. Boxes in the boxplot are ranging from 25th to 75th percentile, with the median represented as a line inside the box. The whiskers of the boxplot extend from both percentiles, 75th percentile for upper whisker and 25th for lower whisker, to the values within 1.5xIQR. IQR is the inter-quartile range, also known as the distance from the first to the third quartile. Data outside the whiskers' ends are outliers and are plotted individually. On the odds ratio plots, middle dot represents odds ratio value for represented IgGP surrounded by its 95% confidence interval (CI). Odds ratio of 1.0 means that there is no

difference between compared groups for the given IgGP. Asterisk symbols represent statistical significance: * <0.05; **<0.01.

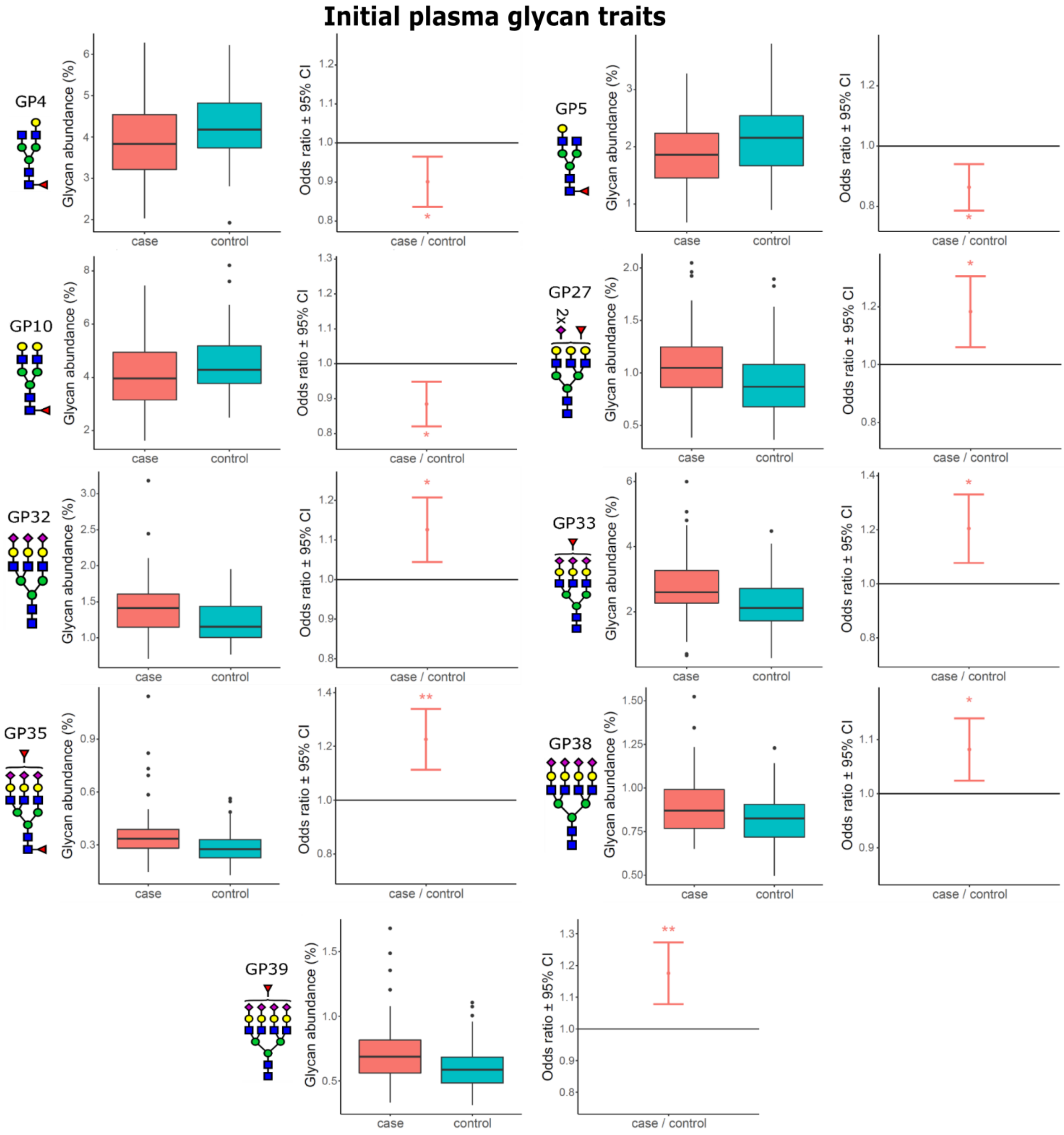

Figure S2 Most prominent differences between MS subjects and matched controls in directly measured plasma protein glycans. The left part of the figure shows boxplots representing differences in glycan abundances, while the right part shows odds ratio plots, resulting from post-hoc tests. Boxes in the boxplot are ranging from 25th to 75th percentile, with the median represented as a line inside the box. The whiskers of the boxplot extend from both percentiles, 75th percentile for upper whisker and 25th for lower whisker, to the values within 1.5xIQR. IQR is the inter-quartile range, also known as the distance from the first to the third quartile. Data outside the whiskers' ends are outliers and are plotted individually. On the odds ratio plots, middle dot represents odds

ratio value for represented GP surrounded by its 95% confidence interval (CI). Odds ratio of 1.0 means that there is no difference between compared groups for the given GP. Asterisk symbols represent statistical significance: * <0.05; **<0.01.

Table S4 Comparison of IgG and plasma protein derived glycan traits between subjects with multiple sclerosis and matched controls. Differences are expressed as odds ratio values, resulting from post-hoc tests. Glycan data were adjusted for age and sex, while false discovery rate was controlled using Benjamini-Hochberg method. Significant differences are in bold.

N-glycan origin	*Derived trait*	*Odds ratio*	*95% CI*	*p-value*	*Adjusted p-value*
**IgG**	**CF**	**0.84**	**0.77 – 0.91**	**7.03 x 10^{-05}**	**6.96 x 10^{-03}**
**IgG**	**HM**	**1.17**	**1.06 – 1.28**	**7.48 x 10^{-04}**	**1.48 x 10^{-02}**
IgG	B	1.07	1.02 – 1.12	8.04 x 10^{-03}	9.95 x 10^{-02}
IgG	S0	0.95	0.90 – 0.99	3.41 x 10^{-02}	1.99 x 10^{-01}
IgG	S2	1.08	1.00 – 1.16	3.25 x 10^{-02}	1.99 x 10^{-01}
IgG	G1	0.97	0.94 – 1.00	5.00 x 10^{-02}	2.18 x 10^{-01}
IgG	G0	1.03	0.94 – 1.12	5.24 x 10^{-01}	7.75 x 10^{-01}
IgG	S1	1.01	0.96 – 1.07	6.45 x 10^{-01}	8.40 x 10^{-01}
IgG	G2	0.99	0.91 – 1.07	7.86 x 10^{-01}	9.05 x 10^{-01}
N-glycan origin	*Derived trait*	*Odds ratio*	*95% CI*	*p-value*	*Adjusted p-value*
**plasma**	**AF**	**1.21**	**1.09 – 1.32**	**1.67 x 10^{-04}**	**6.78 x 10^{-03}**
**plasma**	**LB**	**0.93**	**0.89 – 0.97**	**8.22 x 10^{-04}**	**1.66 x 10^{-02}**
**plasma**	**S3**	**1.09**	**1.04 – 1.15**	**7.06 x 10^{-04}**	**1.66 x 10^{-02}**
**plasma**	**S4**	**1.10**	**1.04 – 1.16**	**8.06 x 10^{-04}**	**1.66 x 10^{-02}**
**plasma**	**G4**	**1.09**	**1.03 – 1.15**	**1.18 x 10^{-03}**	**1.87 x 10^{-02}**
**plasma**	**HB**	**1.08**	**1.03 – 1.13**	**2.16 x 10^{-03}**	**2.19 x 10^{-02}**
**plasma**	**G3**	**1.07**	**1.02 – 1.13**	**5.28 x 10^{-03}**	**4.28 x 10^{-02}**
plasma	G1	0.91	0.85 – 0.98	1.06 x 10^{-02}	7.17 x 10^{-02}
plasma	S1	0.97	0.95 – 1.00	4.10 x 10^{-02}	1.58 x 10^{-01}
plasma	S2	1.03	1.00 – 1.07	6.37 x 10^{-02}	1.95 x 10^{-01}
plasma	CF	0.94	0.88 – 1.00	6.60 x 10^{-02}	1.98 x 10^{-01}
plasma	G0	0.95	0.87 – 1.02	1.83 x 10^{-01}	4.00 x 10^{-01}
plasma	HM	1.01	0.98 – 1.05	3.52 x 10^{-01}	5.66 x 10^{-01}
plasma	G2	0.99	0.95 – 1.02	4.36 x 10^{-01}	6.31 x 10^{-01}
plasma	B	1.01	0.95 – 1.07	7.38 x 10^{-01}	8.54 x 10^{-01}

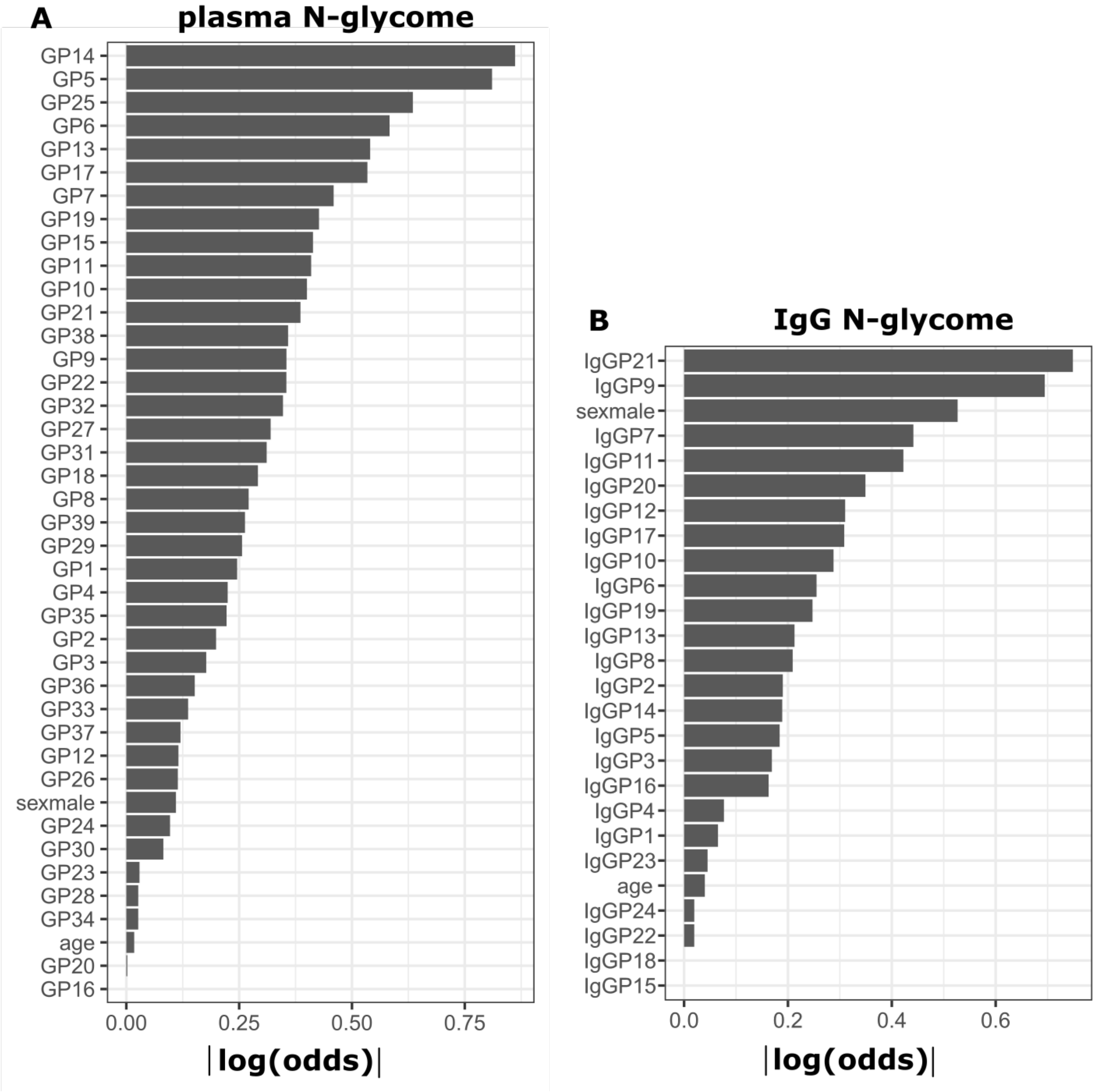

Figure S3 Contribution of individual glycans in multiple sclerosis classification model. Individual glycan performance is expressed as an absolute change in log(odds) for increase of 1 SD in glycan abundance. IgGP – IgG glycan peak; GP – plasma glycan peak
